# New records of powder-post beetles (Coleoptera, Bostrichidae) from China

**DOI:** 10.3897/BDJ.12.e120044

**Published:** 2024-05-22

**Authors:** Yi-Feng Zhang, Ying Wang, Wei Lin, Ling-Zeng Meng

**Affiliations:** 1 College of Biological & Agricultural Sciences, Honghe University, Mengzi, China College of Biological & Agricultural Sciences, Honghe University Mengzi China; 2 Fujian Academy of Forestry, Fuzhou, China Fujian Academy of Forestry Fuzhou China; 3 Animal & Plant Inspection and Quarantine Technology Center of Shenzhen Customs District P.R. China, Shenzhen, China Animal & Plant Inspection and Quarantine Technology Center of Shenzhen Customs District P.R. China Shenzhen China; 4 Shenzhen Academy of Inspection and Quarantine, Shenzhen, China Shenzhen Academy of Inspection and Quarantine Shenzhen China; 5 Technical Center of Gongbei Customs District P. R. China, Zhuhai, China Technical Center of Gongbei Customs District P. R. China Zhuhai China

**Keywords:** Bostrichidae, new records, China

## Abstract

**Background:**

The Bostrichidae is a small family of Coleoptera, with up to 600 described species all over the world. Almost 90 species of the family have been recorded in China at present and several new species of powder-post beetles have been described in recent years. Since the 1940s, the family Bostrichidae has become a neglected group in the taxonomy research of the Chinese mainland compared with its extensive territorial and super complex ecological diversity. Thus, there is need for more field specimen collection and in-depth taxonomic study.

**New information:**

In this study, two powder-post beetles species *Trogoxylonspinifrons* (Lesne, 1910) and *Mintheabivestita* Lesne, 1937, are recorded for the first time from China. New provincial distribution records of another 18 Bostrichidae also listed.

## Introduction

The family Bostrichidae is commonly recognised as a major pest group with an important economical value due to its negative impact on agroforestry (Chu and Zhang 1997, Chen 2011). Almost 90 species of the family have been recorded in China and several new species of powder-post beetles have been described in recent years (Chu and Zhang 1997, Hua 2002, Liu et al. 2006, Borowski 2007, Borowski and Węgrzynowicz 2007, Liu 2010, Chen 2011, Liu 2021, Zhang et al. 2022, Liu and Beaver 2023). However, detailed locality information on some of China's existing Bostrichidae species have not been specified (Chu and Zhang 1997, Borowski 2007, Borowski and Węgrzynowicz 2007, Chen 2011, Liu 2021). China has a vast territory and its geographical span is very large compared to most countries. These faunistic records, which are not recorded in detail at the provincial level, have caused problems for understanding the distribution of the species. In the present study, we examined most of the important collections of Bostrichidae specimens in China and found certain unpublished faunistic records worthwhile to publish. Thus, this study provides a list of Bostrichidae's new records in China to the present time.

## Materials and methods

The majority of the materials of this study come from the first author who examined most of the important collections of Bostrichidae specimens in China during the period from 2021 to 2023. Some other materials were also donations from the author's friend. Most of the basis about the new record in this study were preserved specimens in institutions. Only the new record of *Coccographisnigrorubra* Lesne, 1901 in Anhui and Zhejiang Province were photos from friends. The first author was responsible for all identified and determined materials of the new record.

**Figure 1.**
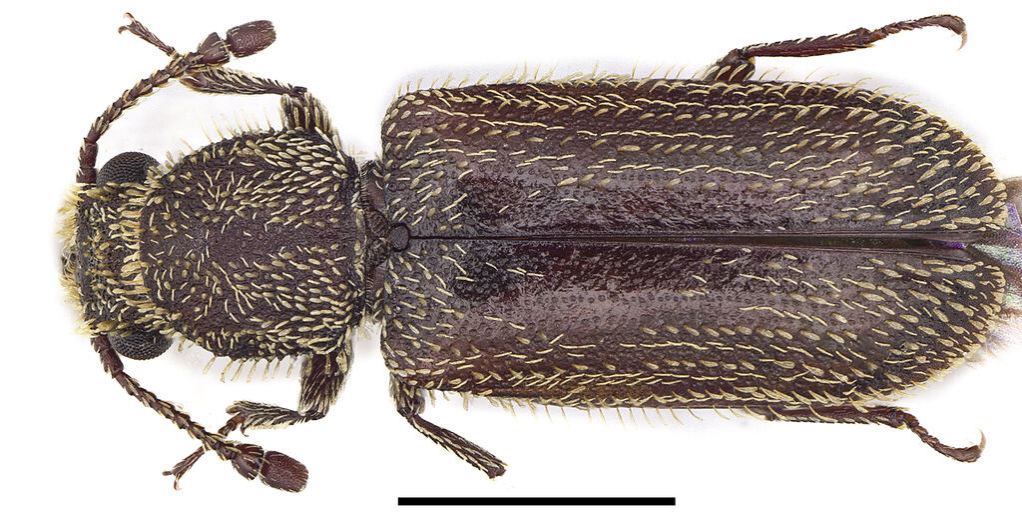
Dorsal view of *Mintheabivestita* Lesne, 1937. Scale bar = 1 mm.

**Figure 2.**
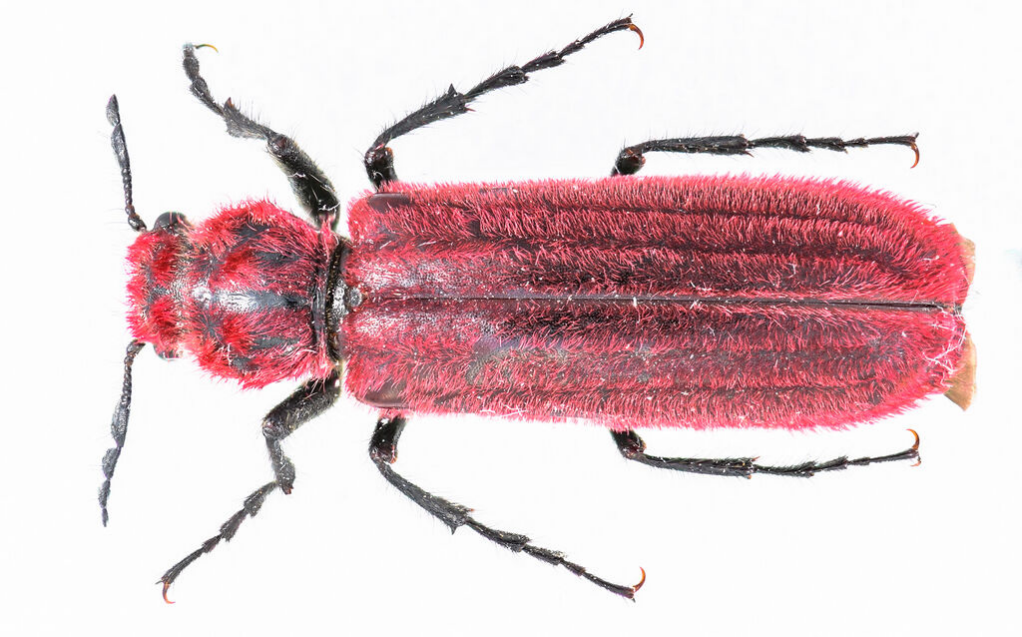
Dorsal view of *Coccographisnigrorubra* Lesne, 1901.

**Figure 4.**
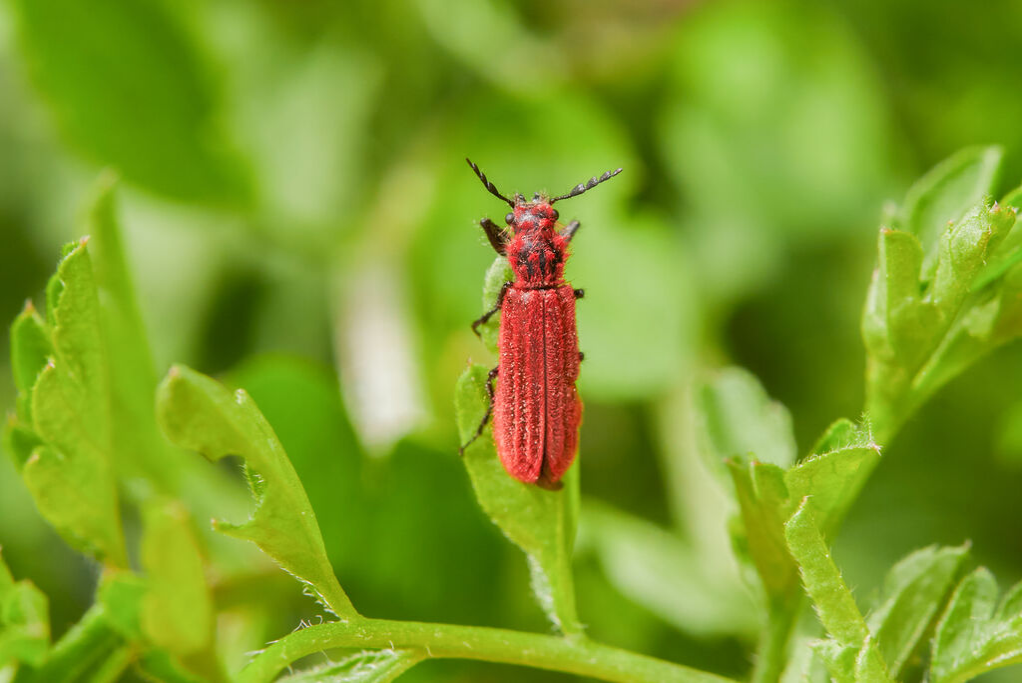
Adults of *Coccographisnigrorubra* Lesne, 1901 on a leaf in Wu-Chao-Shan, Zhejiang at an altitude of 100 m at daytime in March 2024 (photo by Jian-Bo Li).

Fig. 1 and Fig. 2 were taken with a Leica 1DVM6A. Fig. 3 was taken with Canon EOS E6 Mark II & EOS R7 and RF100-400mm f5.6-8 IS USM lens by Wen-Qi Zhu and Jia-Min Chen. Fig. 4 was taken by Jian-Bo Li. Both figures were combined through Helicon Focus 8.2.5 software and optimised with Adobe Photoshop CC 2020.

Abbreviations for the institution having custody of the specimens in this study are:

- **CLYQ** Museum of Chong-Lin-Ye-Qu Cultural Creativity Co., Ltd., Fuzhou, China
- **GCD** Gongbei Customs District P.R. China, Zhuhai, China
- **GIZ** Museum of Institute of Zoology Guangdong Academy of Sciences, Guangzhou, China
- **HU** Honghe University, Mengzi, China
- **IOZ** Institute of Zoology, Chinese Academy of Science, Beijing, China
- **SCD** Shenzhen Customs District P.R. China, Shenzhen, China
- **SYS** The Museum of Biology, Sun Yat-sen University, Guangzou, China
- **ZYF** First author’s private collection, Fuzhou, China

## Checklists

### New country or province records from China

#### Bostrichidae

Latreille, 1802

949FB4B6-BD93-588E-872C-ACC5B7CEF6F2

#### Dinoderinae

C. G. Thomson, 1863

DA48EDC2-5918-5D77-B29E-511E80D063D7

#### Dinoderus

Stephens, 1830

BC56E484-DFA3-512D-B277-1E1D85728967

#### Dinoderus bifoveolatus

(Wollaston, 1858)

24A29A38-3979-52F7-BD68-2F5CDAF2D18A

Rhyzopertha bifoveolata Wollaston, 1858

##### Materials

- **Type status:** Other material. **Occurrence:** individualCount: 1; lifeStage: adult; occurrenceID: 2A1C06F7-5156-5AFF-A06A-ACCC038339BF; **Location:** country: China; stateProvince: Yunnan; county: Xishuangbanna; municipality: Jinghong; locality: Jinuo Mountain; verbatimLocality: 基诺山; verbatimElevation: 1456 m; locationRemarks: label: YN, XSBN, JNS, 22°03′29.66″N, 100°40′47.18″E, 31.05.2022, 1456 m, leg. H.T. Song; **Identification:** identifiedBy: Yi-Feng Zhang; dateIdentified: 02-2023; **Record Level:** institutionCode: HU; basisOfRecord: PreservedSpecimen

##### Distribution

Cosmopolitan.

##### Notes

New record for Yunnan Province. This species previously had only an unspecific record in China (Borowski 2007), with no detailed provincial distribution record.

#### Dinoderus brevis

Horn, 1878

AC517021-8201-510B-A07E-7656FE7FAE87

##### Materials

- **Type status:** Other material. **Occurrence:** individualCount: 1; lifeStage: adult; occurrenceID: 26712712-B833-5882-AE3D-8BB99547C485; **Location:** country: China; stateProvince: Yunnan; county: Xishuangbanna; municipality: Mengla; verbatimLocality: 勐腊; locationRemarks: label: YN, XSBN, Mengla, 14.07.1959, leg. Y.R. Wang, IOZ(E)1034405 | *Dinoderusminutus* det. L.Y. Liu; **Identification:** identifiedBy: Yi-Feng Zhang; dateIdentified: 03-2023; **Record Level:** institutionCode: IOZ; basisOfRecord: PreservedSpecimen

##### Distribution

Cosmopolitan (mostly tropical regions).

##### Notes

New record of Yunnan Province. This species previously had only an unspecific record in China (Borowski 2007), with no detailed provincial distribution record.

#### Dinoderus favosus

Lesne, 1911

BB4346E8-E73F-5F66-94B0-A695B0ADA74F

##### Materials

- **Type status:** Other material. **Occurrence:** individualCount: 1; lifeStage: adult; occurrenceID: AE2BCB87-40C3-5471-AC63-4373F31A4DD7; **Location:** country: China; stateProvince: Guangdong; county: Zhuhai; locality: Fenghuang Mountain; verbatimLocality: 凤凰山长南径古道; verbatimElevation: 20-100 m; locationRemarks: label: GD, ZH, FHS, Changnanjing old road, Host of Gnetumluofuense, 15.05.2023, leg. W. Lin; **Identification:** identifiedBy: Yi-Feng Zhang; dateIdentified: 01-07-2023; **Record Level:** institutionCode: GCD; basisOfRecord: PreservedSpecimen

##### Host of

*Gnetumluofuense* (new host plant species founded in Guangdong).

##### Distribution

China (Yunnan, Guangdong), India (Andaman Islands), Malaysia, Thailand, Vietnam, Myanmar (Borowski and Węgrzynowicz 2007, Zhang et al. 2022, Sittichaya 2023).

##### Notes

New record of Guangdong Province.

#### Bostrichinae

Latreille, 1802

7DECD05C-5D8E-55AD-87D1-50DA7D29EC08

#### Sinoxylini

Lesne, 1899

048BFADC-B93B-5C24-B0B2-7D63BEA215A7

#### Sinoxylon

Duftschmid, 1825

42803965-2BD9-5C23-9E6D-E26B434F5D48

#### Sinoxylon tignarium

Lesne, 1902

776E8473-5D00-5BBA-AC18-1941DB192178

##### Materials

- **Type status:** Other material. **Occurrence:** individualCount: 1; lifeStage: adult; occurrenceID: 03FAABB2-7412-59B6-A363-EFF58A5905E3; **Location:** country: China; stateProvince: Hainan; county: Ledong; locality: Jianfeng Mountain (main peak), Backyard of a Farmhouse restaurant; verbatimLocality: 尖峰岭主峰峰谷农家乐后院; verbatimElevation: 688 m; locationRemarks: label: PYQ351, HN, JFL, farmhouse restaurant backyard 3#, JFL3, 18°41′42.37″N, 108°51′36.11″E, 688 m, 20-29.02.2020, leg. C.Y. Xu; **Identification:** identifiedBy: Yi-Feng Zhang; dateIdentified: 19-07-2023; **Record Level:** institutionCode: GCD; basisOfRecord: PreservedSpecimen
- **Type status:** Other material. **Occurrence:** individualCount: 1; lifeStage: adult; occurrenceID: 68FC6434-0991-5120-A26F-BB69A5BD6D0C; **Location:** country: China; stateProvince: Guangdong; county: Shaoguan; municipality: Qujiang; locality: Luokeng; verbatimLocality: 罗坑; verbatimElevation: 140-240 m; locationRemarks: label: GD, SG, LK, 10-17.07.2023, leg. W. Lin; **Identification:** identifiedBy: Yi-Feng Zhang; dateIdentified: 19-07-2023; **Record Level:** institutionCode: GCD; basisOfRecord: PreservedSpecimen
- **Type status:** Other material. **Occurrence:** individualCount: 1; lifeStage: adult; occurrenceID: F16C1131-A9E6-5EA5-9DE5-7D4A707269F6; **Location:** country: China; stateProvince: Guangdong; county: Shenzhen; municipality: Nanshan; locality: Shekou; verbatimLocality: 蛇口; verbatimElevation: 10 m; locationRemarks: label: GD, SZ, SK, 10.07.2023, leg. Y. Wang; **Identification:** identifiedBy: Yi-Feng Zhang; dateIdentified: 20-07-2023; **Record Level:** institutionCode: SCD; basisOfRecord: PreservedSpecimen

##### Distribution

China (Sichuan, Yunnan, Guangdong, Hainan), Sri Lanka, north-eastern India, Vietnam, Thailand (Borowski 2007, Chen 2011, Liu and Beaver 2018).

##### Notes

New record of Guangdong and Hainan Province.

#### Bostrichini

Latreille, 1802

59D7ED97-C223-5D2C-8287-D3E3798F621D

#### Heterobostrychus

Lesne, 1899

82DBCF03-5CB2-5ADB-B768-21E1753EFE29

#### Heterobostrychus pileatus

Lesne, 1899

4964DB6D-9480-57C3-993E-77D249833957

##### Materials

- **Type status:** Other material. **Occurrence:** individualCount: 2; sex: 1 male, 1 female; lifeStage: adult; occurrenceID: E8934ED3-519C-53A7-8536-C669AB331258; **Location:** country: China; stateProvince: Guangxi; county: Laibin; municipality: Jinxiu; locality: Toupai, at the foot of Yuehuang Mountain; verbatimLocality: 头排镇月皇岭脚下; verbatimElevation: 100 m; locationRemarks: label: GX, LB, DYS, 100 m, 15.05.2023, Light trap, leg. C.F. Feng; **Identification:** identifiedBy: Yi-Feng Zhang; dateIdentified: 23-05-2023; **Record Level:** institutionCode: HU; basisOfRecord: PreservedSpecimen

##### Distribution

China (Yunnan, Guangxi), India, Nepal, Myanmar, Thailand, Laos, Cambodia, Vietnam, Philippines (Borowski and Węgrzynowicz 2007, Liu and Beaver 2018).

##### Notes

New record of Guangxi Province.

#### Parabostrychus

Lesne, 1899

A31CC3BD-11E3-5C34-A565-B0B46D486046

#### Parabostrychus elongatus

(Lesne, 1895)

9903BEB0-0BF0-5F83-BEDF-6529279AFA21

Bostrychus elongatus Lesne, 1895

##### Materials

- **Type status:** Other material. **Occurrence:** individualCount: 1; lifeStage: adult; occurrenceID: 0FB791A1-04A6-5258-A4AE-C189BB6EBA98; **Location:** country: China; stateProvince: Guangdong; county: Dongguan; locality: Daling Mountain; verbatimLocality: 大岭山; verbatimElevation: 110 m; locationRemarks: label: LSX485 GD, DG, DLS, GD5 Ma, 22°51'52.79"N, 113°44'46.01"E, 28.02-30.03.2022, 110 m, leg. L.L. Chen et al.; **Identification:** identifiedBy: Yi-Feng Zhang; dateIdentified: 02-02-2023; **Record Level:** institutionCode: GCD; basisOfRecord: PreservedSpecimen

##### Distribution

China (Yunnan, Guangdong, Sichuan, Hubei), India, Vietnam (Liu and Beaver 2018, Liu 2021, Zhang et al. 2022).

##### Notes

New record of Guangdong Province.

#### Parabostrychus acuticollis

Lesne, 1913

DDC7E233-FECA-5B64-B0A7-8741FB9F06F4

##### Materials

- **Type status:** Other material. **Occurrence:** individualCount: 1; lifeStage: adult; occurrenceID: 1191F2AD-CDC4-5B41-8CB7-E0885FCC6AA5; **Location:** country: China; stateProvince: Guangdong; county: Shaoguan; municipality: Qujiang; locality: Guangdong Luokeng *Shinisauruscrocodilurus* National Nature Reserve; verbatimLocality: 鳄蜥保护区; verbatimElevation: 600-800 m; locationRemarks: label: GD, SG, LK, *Shinisauruscrocodilurus* Nature Reserve, 08.04.2023, leg. W. Lin; **Identification:** identifiedBy: Yi-Feng Zhang; dateIdentified: 19-07-2023; **Record Level:** institutionCode: GCD; basisOfRecord: PreservedSpecimen

##### Distribution

China (Shanghai, Jiangsu, Hubei, Anhui, Sichuan, Beijing, Taiwan, Hunan, Shandong, Yunnan, Zhejiang, Guangdong), India, Nepal, Thailand (Chu and Zhang 1997, Liu et al. 2006, Liu and Beaver 2018, Zhang et al. 2022).

##### Notes

New record of Guangdong Province.

#### Micrapate

Casey, 1898

46C5C7C9-DEA8-58A5-A9F2-EFE86F358EF3

#### Micrapate simplicipennis

(Lesne, 1895)

44BDF170-4546-5033-9AA5-AD5D3256B3F4

Xylopertha simplicipennis Lesne, 1895

##### Materials

- **Type status:** Other material. **Occurrence:** individualCount: 10; lifeStage: adult; occurrenceID: 42DFA292-5453-5B00-8BC5-ED6FC3DC9DE2; **Location:** country: China; stateProvince: Guangxi; county: Beihai; municipality: Hepu; locality: Baisha Youhang Village; verbatimLocality: 白沙 油行村; verbatimElevation: 20-50 m; locationRemarks: label: GX, HP, Youhangchun, Host of *Meliaazedarach*, 19.01.2023, leg. W. Lin; **Identification:** identifiedBy: Yi-Feng Zhang; dateIdentified: 02-02-2023; **Record Level:** institutionCode: GCD; basisOfRecord: PreservedSpecimen
- **Type status:** Other material. **Occurrence:** individualCount: 1; lifeStage: adult; occurrenceID: 93A6B5DD-5C4F-55EC-9024-7C8D70463B5E; **Location:** country: China; stateProvince: Hainan; county: Baisha; municipality: Nankai; locality: forest of *Areca*; verbatimLocality: 槟榔林; verbatimElevation: 265 m; locationRemarks: label: PYQ351, HN, NKX, forest of Areca, HN11 Ma, 19°4′44.68″N, 109°24′4.74″E, 265 m, 31.03.-30.04.2022, leg. L.L. Chen; **Identification:** identifiedBy: Yi-Feng Zhang; dateIdentified: 02-02-2023; **Record Level:** institutionCode: GCD; basisOfRecord: PreservedSpecimen

##### Host of

*Meliaazedarach* (new host plant found in Guangxi).

##### Distribution

China (Yunnan, Chongqing, Zhejiang, Guangxi, Hainan), India, Indonesia, Laos, Myanmar, Nepal, Thailand, Vietnam (Liu and Beaver 2018, Liu 2021).

##### Notes

New record of Guangxi and Hainan Provinces.

#### Xyloperthini

Lesne, 1921

67D6C6B5-28C4-5007-8005-3204387AFA7A

#### Xylopsocus

Lesne, 1901

BDFF8070-74F6-5E54-AA3A-93D072ED1315

#### Xylopsocus acutespinosus

Lesne, 1906

6017F77B-AF41-5B21-BE69-350AA2691905

##### Materials

- **Type status:** Other material. **Occurrence:** individualCount: 1; lifeStage: adult; occurrenceID: 6C676D3F-9357-5D63-B7B9-3F0758F61BE1; **Location:** country: China; stateProvince: Xizang; county: Linzhi; municipality: Motuo; locality: Beibeng; verbatimLocality: 背崩乡; verbatimElevation: 800 m; locationRemarks: label: XZ, MT, BB, Light trap, N29.24305°, E95.17038°, 800 m, 07.08.2017, leg. R. Zhou; **Identification:** identifiedBy: Yi-Feng Zhang; dateIdentified: 03-2023; **Record Level:** institutionCode: IOZ; basisOfRecord: PreservedSpecimen

##### Distribution

China (Shaanxi, Yunnan, Xizang), India, Laos, Myanmar, Nepal, Thailand (Chu and Zhang 1997, Liu and Beaver 2018, Liu 2021).

##### Notes

New record of Xizang Autonomous Region.

#### Xylopsocus radula

Lesne, 1901

CCE2E1B9-EA88-5A7D-A1AB-0EA30F651E9D

##### Materials

- **Type status:** Other material. **Occurrence:** individualCount: 1; lifeStage: adult; occurrenceID: 32E97B15-520A-5E8E-B6A4-AB33BA185F75; **Location:** country: China; stateProvince: Guangdong; county: Shaoguan; municipality: Qujiang; locality: Luokeng Reservoir; verbatimLocality: 罗坑水库; verbatimElevation: 150 m; locationRemarks: label: GD, SG, LKSK, 24°32′9″N, 113°23′26″E, 150 m, 13.03.2022, leg. Y.L. Liao; **Identification:** identifiedBy: Yi-Feng Zhang; dateIdentified: 02-02-2023; **Record Level:** institutionCode: GCD; basisOfRecord: PreservedSpecimen

##### Distribution

China (Yunnan, Guangdong), India, Indonesia, Myanmar, Thailand, Malaysia (Beaver et al. 2011, Liu 2021).

##### Notes

New record of Guangdong Province.

#### Paraxylion

Lesne, 1941

02B438A9-BC5A-5936-BBD5-5AC3C47D5482

#### Paraxylion bifer

(Lesne, 1932)

76467D36-7441-51B7-B9CF-2275BCAF9B1A

Xylion bifer Lesne, 1932

##### Materials

- **Type status:** Other material. **Occurrence:** individualCount: 1; sex: male; lifeStage: adult; occurrenceID: A2AB54FB-B889-5399-A8AC-081E355E9AB6; **Location:** country: China; stateProvince: Hainan; county: Wuzhishan; municipality: Tongshi; verbatimLocality: 通什; locationRemarks: label: HN, Tongshi, 08.06.1983, leg. M.H. Ke; **Identification:** identifiedBy: Yi-Feng Zhang; dateIdentified: 01-02-2023; **Record Level:** institutionCode: GIZ; basisOfRecord: PreservedSpecimen
- **Type status:** Other material. **Occurrence:** individualCount: 2; sex: 2 female, 2 male; lifeStage: adult; occurrenceID: 71860D6B-99ED-5B24-ABDD-2A27F1EF78B0; **Location:** country: China; stateProvince: Hunan; county: Huaihua; municipality: Huitong; locality: Tuanhe Diaotang Village; verbatimLocality: 团河 吊塘村; locationRemarks: label: HN, HT, TH, DT, 13.07.2023, Light trap, leg. J.H. Huang; **Identification:** identifiedBy: Lin Wei; dateIdentified: 31-08-2023; **Record Level:** institutionCode: GCD; basisOfRecord: PreservedSpecimen

##### Distribution

China (Hong Kong, Yunnan, Guangdong, Hainan, Hunan), India, Indonesia, Myanmar, Laos, Vietnam, Malaysia, Thailand (Liu and Beaver 2018, Zhang et al. 2022, Sittichaya 2023).

##### Notes

New record of Hunan and Hainan Province.

#### Xylocis

Lesne, 1901

C59C528D-6638-55FB-B97C-8781D338A247

#### Xylocis tortilicornis

Lesne, 1901

99EC9B75-D939-50F1-A21F-9CA0E0D5F946

##### Materials

- **Type status:** Other material. **Occurrence:** individualCount: 18; sex: 9 female, 9 male; lifeStage: adult; occurrenceID: C68F7140-409E-5AE2-A9B0-FF2923BAFE7F; **Location:** country: China; stateProvince: Guangxi; county: Beihai; municipality: Hepu; locality: Baisha Youhang Village; verbatimLocality: 白沙 油行村; verbatimElevation: 20-50 m; locationRemarks: label: GX, HP, Youhangchun, Host of Ficushispida, 22.01.2023, leg. W. Lin; host of Ficushispida; **Identification:** identifiedBy: Yi-Feng Zhang; dateIdentified: 02-02-2023; **Record Level:** institutionCode: GCD; basisOfRecord: PreservedSpecimen
- **Type status:** Other material. **Occurrence:** individualCount: 1; sex: male; lifeStage: adult; occurrenceID: AA61CA67-8E66-5977-AFEE-D7245AFDBF86; **Location:** country: China; stateProvince: Hainan; locationRemarks: label: HN, 22.06.1974, IOZ(E)1034265 | *Xylocistortilicornis* det. L.Y. Liu; **Record Level:** institutionCode: IOZ; basisOfRecord: PreservedSpecimen

##### Host of

*Ficushispida* (new host plant species found in Guangxi).

##### Distribution

China (Yunnan, Hong Kong, Taiwan, Guangxi, Hainan, Fujian), India, Nepal, Sri Lanka, Laos, Thailand (Liu et al. 2006, Liu and Beaver 2018, Liu 2021).

##### Notes

New record of Guangxi and Hainan Provinces.

#### Apatini

Jacqueln du Val, 1861 sensu Liu & Schönitzer

C81AFA6D-AD72-5A47-B87F-70D3783641FB

#### Phonapate

Lesne, 1895

B91C08E2-3CEE-5339-8C38-9A978ADB330A

#### Phonapate fimbriata

Lesne, 1909

E53B21BA-105B-598C-99DE-F87E02C80064

##### Materials

- **Type status:** Other material. **Occurrence:** individualCount: 1; sex: female; lifeStage: adult; occurrenceID: F3D5913C-1FAD-583E-A040-54EFF9AE9F3D; **Location:** country: China; stateProvince: Fujian; county: Zhangzhou; municipality: Yunxiao; locality: Zhuta Village; verbatimLocality: 竹塔村; verbatimElevation: 12 m; locationRemarks: label: FJ, ZZ, YX, ZT, 23°55'11.77"N, 117°24'45.53"E, 12 m, 27.08.2023, Light trap, leg. Y.F. Zhang; **Identification:** identifiedBy: Yi-Feng Zhang; dateIdentified: 27-08-2023; **Record Level:** institutionID: ZYF; basisOfRecord: PreservedSpecimen
- **Type status:** Other material. **Occurrence:** individualCount: 1; sex: male; lifeStage: adult; occurrenceID: E78E090C-4C98-5CEC-AC44-D8EA68005A20; **Location:** country: China; stateProvince: Jiangxi; county: Jinggangshan; locality: Xiangzhou; verbatimLocality: 湘洲; locationRemarks: label: JX, JGS, XZ 26.iv.2011 leg. Y. Mei, L.-J. Yang, Y.-L. Yu, P. Liu; En-363179 SYS; Phonapatefimbriata (male) det. Y.F. Zhang, 2021; **Identification:** identifiedBy: Yi-Feng Zhang; dateIdentified: 24-08-2021; **Record Level:** institutionCode: SYS; basisOfRecord: PreservedSpecimen
- **Type status:** Other material. **Occurrence:** individualCount: 1; sex: female; lifeStage: adult; occurrenceID: 687AA079-1D84-52B9-A8BA-BF7B61FD34C5; **Location:** country: China; stateProvince: Guangdong; county: Zhaoqing; municipality: Fengkai; locality: Hei-Shi-Ding; verbatimLocality: 黑石顶; locationRemarks: label: GD, FK, HSD 02-vi-2011 (light trap) leg. Y. Li; En-344048 SYS; Phonapatefimbriata (female) det. Y.F. Zhang, 2021; **Identification:** identifiedBy: Yi-Feng Zhang; dateIdentified: 24-08-2021; **Record Level:** institutionCode: SYS; basisOfRecord: PreservedSpecimen

##### Host of

*Litchichinensis* (Lu et al. 2012).

##### Distribution

China (Yunnan, Guangxi, Guangdong, Hong Kong, Jiangxi, Fujian), India, Vietnam, Indonesia, Thailand (Chu and Zhang 1997, Borowski and Węgrzynowicz 2007, Beaver et al. 2011, Lu et al. 2012).

##### Notes

New record of Guangdong, Jiangxi and Fujian Provinces. Liu (2021) mis-listed Yunnan (Original text: "YN") as Hunan (Original text: "HN") from Chen (2011) and neglect Guangxi which had been recorded in Lu et al. (2012)

#### Lyctinae

Billberg, 1820

C3245DB2-CEAA-51D9-B9D0-5C2A87FF8DDB

#### Lyctini

Billberg, 1820

63FB2534-1DD8-5B73-B478-1F7A5B9D4420

#### Lyctus

Fabricius, 1792

D4A511B4-F0FB-5196-BFF9-EA4275118A2F

#### Lyctus africanus

Lesne, 1907

5B43134C-6BB9-571B-948C-EF893357CCBE

##### Materials

- **Type status:** Other material. **Occurrence:** individualCount: 1; occurrenceID: 28744273-A0EA-5EBD-B574-6A7F54B6E697; **Location:** country: China; stateProvince: Hainan; county: Ledong; locality: Jianfeng Mountain; verbatimLocality: 尖峰; locationRemarks: label: HN, JF, Host of *Heveabrasiliensis*, 20.02.1979, determined by Unknown; **Record Level:** institutionCode: IOZ; basisOfRecord: PreservedSpecimen

##### Host of

*Heveabrasiliensis* (Beaver et al. 2011).

##### Distribution

China (Zhejiang, Guangdong, Guangxi, Sichuan, Yunnan, Hainan) (Hua 2002). This is a rather pan-tropical species trending towards a cosmopolitan distribution (Liu 2021).

##### Notes

New record of Hainan Province.

#### Lyctoxylon

Reitter, 1879

C242DDD9-1278-5B4C-8E12-AC46BCDD08B2

#### Lyctoxylon dentatum

(Pascoe, 1866)

CD5B4A64-38B0-5602-ADD6-AE3AB96B0CC9

Minthea dentata Pascoe, 1866

##### Materials

- **Type status:** Other material. **Occurrence:** individualCount: 1; lifeStage: adult; occurrenceID: 26C74B44-B27C-5005-8E6F-B3A8DA4D7690; **Location:** country: China; stateProvince: Hainan; county: Ledong; locality: Jianfeng Mountain; verbatimLocality: 尖峰; locationRemarks: label: HN, JF, Host of *Albiziaprocera*, 15.08.1979, determined by Unknown; **Record Level:** institutionCode: IOZ; basisOfRecord: PreservedSpecimen

##### Host of

*Albiziaprocera* (new host plant found in Hainan).

##### Distribution

China (Guangxi, Guizhou, Hubei, Yunnan, Zhejiang, Jiangxi, Taiwan, Hainan), India, Thailand, Malay Peninsula, Vietnam, Indonesia, Japan, Australia (Hua 2002, Liu and Geis 2019, Liu 2021).

##### Notes

New record of Hainan Province.

#### Minthea

Pascoe, 1866

9D8AF7AE-BE76-5B3E-B30C-1FF8F467B363

#### Minthea bivestita

Lesne, 1937

91EF81AF-76E5-514D-B23A-3FAC9974AD0B

##### Materials

- **Type status:** Other material. **Occurrence:** individualCount: 1; lifeStage: adult; occurrenceID: 543B50DF-417E-58D1-B2C7-3B31F8129306; **Location:** country: China; stateProvince: Yunnan; county: Xishuangbanna; municipality: Jinghong; locality: Shenshi Mountain; verbatimLocality: 神石山; verbatimElevation: 1456 m; locationRemarks: label: YN, XSBN, SSS, 22°04′01.54″N, 100°40′50.52″E, 11.05.2022, 1456 m, leg. H.T. Song; **Identification:** identifiedBy: Yi-Feng Zhang; dateIdentified: 19-07-2023; **Record Level:** institutionCode: GCD; basisOfRecord: PreservedSpecimen

##### Distribution

China (Yunnan), India, Vietnam (Borowski and Węgrzynowicz 2007).

##### Notes

New record of China (Fig. 1).

#### Trogoxylini

Lesne, 1921

6E2EEF72-DC89-559F-AEF4-9755B74AB901

#### Trogoxylon

LeConte, 1862

54950104-5E96-5BF7-A632-0A74EA1E9EE7

#### Trogoxylon impressum

(Comolli, 1837)

48D7E073-5F26-5EF3-94DE-ED91A33F4267

Lyctus impressum Comolli, 1837

##### Materials

- **Type status:** Other material. **Occurrence:** individualCount: 1; lifeStage: adult; occurrenceID: 6F87EFB2-CFB0-56C7-8EA4-EA8970C2FAFD; **Location:** country: China; stateProvince: Hainan; county: Lingshui; verbatimLocality: 陵水; locationRemarks: label: HN, LS, 06.06.1957, leg. T.M. Liu | Trogoxylonimpressum det. L.Y. Liu, 2019; **Identification:** identifiedBy: Lan-Yu Liu; dateIdentified: 2019; **Record Level:** institutionCode: IOZ; basisOfRecord: PreservedSpecimen
- **Type status:** Other material. **Occurrence:** individualCount: 1; lifeStage: adult; occurrenceID: 679F39FA-D90B-5442-A477-54D09857D950; **Location:** country: China; stateProvince: Shandong; county: Qingdao; municipality: Jimo; verbatimLocality: 即墨; locationRemarks: label: SD, JX, 02.06.1958 Host of wheat & pea; **Identification:** identifiedBy: Yi-Feng Zhang; dateIdentified: 03-2023; **Record Level:** institutionCode: IOZ; basisOfRecord: PreservedSpecimen
- **Type status:** Other material. **Occurrence:** individualCount: 1; lifeStage: adult; occurrenceID: 503FF89A-E559-5804-B67E-B4123133596E; **Location:** country: China; stateProvince: Qinghai; county: Haidong; municipality: Minhe; verbatimLocality: 民和; locationRemarks: label: QH, MH, 01.07.1959; **Identification:** identifiedBy: Yi-Feng Zhang; dateIdentified: 03-2023; **Record Level:** institutionCode: IOZ; basisOfRecord: PreservedSpecimen

##### Distribution

China (Hainan, Shandong, Qinghai), Europe, Caucasus, Mediterranean, Turkmenistan, Israel, Iran, Japan and introduced to Australia, New Zealand (Borowski and Węgrzynowicz 2007, Liu and Geis 2019).

##### Notes

New record of Hainan, Shandong Province and Qinghai Autonomous Region. This species previously had only an unspecific record in China (Liu and Geis 2019), with no detailed provincial distribution record. Due to its age, the label information is not detailed. These records may come from an indoor warehousing environment and were imported to the area along with the transportation of host plants (grain), so may be not distributed in these three provinces in an outdoor environment.

#### Trogoxylon spinifrons

(Lesne, 1910)

7D40F0A5-CB56-5EB8-81F6-EFBF8369FBE5

Lyctus spinifrons Lesne 1910

##### Materials

- **Type status:** Other material. **Occurrence:** individualCount: 1; lifeStage: adult; occurrenceID: B3467E52-19D3-52DD-9742-346A99227541; **Location:** country: China; stateProvince: Yunnan; county: Xishuangbanna; municipality: Jinghong; locality: east of Xiaomengyang, in front of a hill slope, on shrubs; verbatimLocality: 小勐养东灌木丛中，山坡前; verbatimElevation: 850 m; locationRemarks: label: YN, XSBN, XMY, 850 m, 18.06.1957, leg. S.Y. Wang | Lyctus sp. 1976.XI | *Trogoxylonspinfrons* (Lesne, 1910) det. L.Y. Liu; **Identification:** identifiedBy: Lan-Yu Liu; dateIdentified: 2019; **Record Level:** institutionCode: IOZ; basisOfRecord: PreservedSpecimen

##### Distribution

China (Yunnan), India, Vietnam, Papua New Guinea, Thailand (Borowski and Węgrzynowicz 2007, Liu 2010).

##### Notes

New record of China.

#### Polycaoninae

Lesne, 1896

B6507742-594D-536A-8F9E-946EF44EA7C3

#### Melalgus

Dejean, 1833

1775CC8F-1268-5574-BA2A-E24705C7F06B

#### Melalgus batillus

(Lesne, 1902)

024AC002-75A2-5857-BE86-6C2E32558160

Heterarthron batillum Lesne, 1902

##### Materials

- **Type status:** Other material. **Occurrence:** individualCount: 1; sex: male; lifeStage: adult; occurrenceID: 062BD6A6-9541-5DE7-A70D-D3EABA1D8E3E; **Location:** country: China; stateProvince: Guangxi; county: Fangchenggang; municipality: Fangcheng; locality: Dalu, Shanzhong Village; verbatimLocality: 大菉镇山中村山中保护站; verbatimElevation: 69 m; locationRemarks: label: GX, FCG, DL, SZC, 18.03.2021, Light trap, 21.8141°N, 108.1242°E, 69 m, leg. H. Liu & K.Y. Zhang; **Identification:** identifiedBy: Yi-Feng Zhang; dateIdentified: 03-2023; **Record Level:** institutionCode: IOZ; basisOfRecord: PreservedSpecimen
- **Type status:** Other material. **Occurrence:** individualCount: 1; sex: female; lifeStage: adult; occurrenceID: E102EBF1-4C44-5578-959D-FDDA27F6FFF8; **Location:** country: China; stateProvince: Fujian; county: Fuzhou; locality: Forest Park; verbatimLocality: 福州国家森林公园; locationRemarks: label: FJ, Forest Park, 05.06.2002, leg. X.Y. He; **Identification:** identifiedBy: Yi-Feng Zhang; dateIdentified: 2023; **Record Level:** institutionCode: HU; basisOfRecord: PreservedSpecimen
- **Type status:** Other material. **Occurrence:** individualCount: 1; sex: male; lifeStage: adult; occurrenceID: 15FE40BE-5D57-54F6-89B7-00C3E4AF231A; **Location:** country: China; stateProvince: Guangdong; county: Guangzhou; locality: Luo-Gang-Dong; verbatimLocality: 罗岗垌; locationRemarks: label: GD, LGD, 21.V.1963, leg. Shun-Bang Liu; En-080361 SYS; Melalgusbatillus (male) det. Y.-F. Zhang 2021.VIII; **Identification:** identifiedBy: Yi-Feng Zhang; dateIdentified: 24-08-2021; **Record Level:** institutionCode: SYS; basisOfRecord: PreservedSpecimen
- **Type status:** Other material. **Occurrence:** individualCount: 1; sex: female; lifeStage: adult; occurrenceID: E9D64099-EEF7-510E-8D75-2F0BE5DB08FC; **Location:** country: China; stateProvince: Guangdong; county: Shaoguan; municipality: Qujiang; locality: Long-Tou Mountain; verbatimLocality: 龙头山; locationRemarks: label: GD, QJ, LTS VI, 1947; En-080994 SYS; Melalgus Batillus (female) dei. Y.F. Zhang, 2021.08; **Identification:** identifiedBy: Yi-Feng Zhang; dateIdentified: 24-08-2021; **Record Level:** institutionCode: SYS; basisOfRecord: PreservedSpecimen

##### Distribution

China (Yunnan, Hainan, Guangxi, Guangdong, Fujian, Zhejiang, Hong Kong), India, Vietnam, Japan (Liu 2021, Yoshitomi 2021, Zhang et al. 2022, Borowski 2022).

##### Notes

New record of Guangxi, Guangdong and Fujian Provinces.

#### Psoinae

Blanchard, 1851

E866D291-7BFB-5D9F-BF70-8C2758168B50

#### Coccographis

Lesne, 1901

3DB1609B-A918-59D3-868B-6EFF137DE993

#### Coccographis nigrorubra

Lesne, 1901

15AB9715-27D8-5D20-9B6B-A5A2BDFC6B2D

##### Materials

- **Type status:** Other material. **Occurrence:** individualCount: 1; sex: male; lifeStage: adult; occurrenceID: 1E81AC14-F670-5330-A1E9-7FB7BE097B1A; **Location:** country: China; stateProvince: Guangdong; county: Shaoguan; municipality: Qujiang; locality: Luokeng; verbatimLocality: 罗坑; verbatimElevation: 250 m; locationRemarks: label: GD, SG, QJ, LK, 24°29′54″N, 113°20′35″E, 250 m, 13.III.2022, leg. Y.L. Liao; **Identification:** identifiedBy: Yi-Feng Zhang; dateIdentified: 02-02-2023; **Record Level:** institutionCode: GCD; basisOfRecord: PreservedSpecimen
- **Type status:** Other material. **Occurrence:** individualCount: 1; sex: male; lifeStage: adult; occurrenceID: 53817790-51C0-5676-96F1-ECF091165447; **Location:** country: China; stateProvince: Anhui; county: Tongling; municipality: Yian; locality: Feng-Huang-Shan; verbatimLocality: 凤凰山; verbatimElevation: 100-350m; locationRemarks: label: AH, TL, YA, FHS, 31.III.2024, photo by Wen-Qi Zhu and Jia-Min Cheng; **Identification:** identifiedBy: Yi-Feng Zhang; dateIdentified: 01-04-2024; **Record Level:** basisOfRecord: HumanObservation
- **Type status:** Other material. **Occurrence:** individualCount: 1; sex: male; lifeStage: adult; occurrenceID: FF001B4D-495C-5E42-ABA7-5DBE54C2E5B6; **Location:** country: China; stateProvince: Zhejiang; county: Hangzhou; municipality: Yuhang; locality: Wu-Chao-Shan; verbatimLocality: 午潮山; verbatimElevation: 100m; locationRemarks: label: zhejiang-Hangzhou-wuchaoshan-alt. 100m-22.III.2024-photo by Jian-Bo Li; **Identification:** identifiedBy: Yi-Feng Zhang; dateIdentified: 02-04-2024; **Record Level:** basisOfRecord: HumanObservation
- **Type status:** Other material. **Occurrence:** individualCount: 1; sex: male; lifeStage: adult; occurrenceID: 3147DB2E-B22B-52CE-BC8F-309DC0EA4B5A; **Location:** country: China; stateProvince: Fujian; county: Fuzhou; municipality: Yongtai; locality: Tengshan Provincial Nature Reserve, Dong-Hu-Jian; verbatimLocality: 藤山省级自然保护区, 东湖尖; verbatimElevation: 1100m; locationRemarks: label: FJ FZ YT TSZRBHQ DHJ 1100m 26-III-2024 leg. Xing Hu, Liang Guo, Zu-Bin Chen, Qin-Ang Wu; Coccographisnigrorubra Lesne, 1901 (male) det. YF Zhang, 2024; **Identification:** identifiedBy: Yi-Feng Zhang; dateIdentified: 30-03-2024; **Record Level:** institutionCode: CLYQ; basisOfRecord: PreservedSpecimen

##### Distribution

China (Yunnan, Guangdong, Anhui, Zhejiang and Fujian), Vietnam, Laos (Liu 2021).

##### Notes

New record of Guangdong, Anhui, Zhejiang and Fujian Provinces (Fig. 2, Fig. 3 and Fig. 4).

## Discussion

From the Founding of New China until the early 1990s, most of the research reports related to Chinese Bostrichidae beetles focused on their occurrence, prevention and quarantine in indoor storage environments. Relatively few studies have been conducted on outdoor environments, especially forest environments (Xin and Ding 1958, Chen and Liu 1964, GFRI 1977, Shi and Tan 1980, FDY and IOZ 1987). We found that the distribution records of Bostrichidae in China have been affected by previous insufficient research, leading to some mismatches. There are primarily two situations: first, indiscriminate records of indoor storage and outdoor natural environments within China have resulted in literature-recorded distribution ranges far exceeding the natural distribution range of the species; second, due to inadequate field collection and survey efforts, the recorded existing distribution is far smaller than the actual distribution range.

The best example of the first situation is *Rhyzoperthadominica*. According to the latest records, this species is documented as being distributed in almost all provinces and municipalities of China, except for Xinjiang and Xizang, ranging from Heilongjiang in the north to Guangdong in the south (Hua 2002). However this species tends to favour tropical and subtropical climates in outdoor natural environments (Wakil 2014), suggesting that its natural distribution in China should be limited to the southern part. Records from northern regions may likely originate from occurrences in storage facilities.

The best example of the second situation is *Coccographisnigrorubra*. As of 2021, the distribution range of this species is only documented in the region encompassing Vietnam, Laos and Yunnan Province in China (Liu 2021). However, we have found that this species can be distributed as far east as Zhejiang Province and as far north as Anhui Province in China.

In summary, it is urgently required to conduct more extensive and in-depth research on Bostrichidae fauna in China. For future work, it is hoped that specialists will record detailed locality information and distinguish between indoor and outdoor environments when reporting Bostrichidae specimens. Additionally, it is hoped that future research will record more biological information about Bostrichidae, such as host plants, species reproduction and the duration of occurrences. This will aid in understanding the phylogenetic relationships, evolution and the inter-relationships between these species and their environment in comparison to other closely-related taxa.

## Supplementary Material

XML Treatment for Bostrichidae

XML Treatment for Dinoderinae

XML Treatment for Dinoderus

XML Treatment for Dinoderus bifoveolatus

XML Treatment for Dinoderus brevis

XML Treatment for Dinoderus favosus

XML Treatment for Bostrichinae

XML Treatment for Sinoxylini

XML Treatment for Sinoxylon

XML Treatment for Sinoxylon tignarium

XML Treatment for Bostrichini

XML Treatment for Heterobostrychus

XML Treatment for Heterobostrychus pileatus

XML Treatment for Parabostrychus

XML Treatment for Parabostrychus elongatus

XML Treatment for Parabostrychus acuticollis

XML Treatment for Micrapate

XML Treatment for Micrapate simplicipennis

XML Treatment for Xyloperthini

XML Treatment for Xylopsocus

XML Treatment for Xylopsocus acutespinosus

XML Treatment for Xylopsocus radula

XML Treatment for Paraxylion

XML Treatment for Paraxylion bifer

XML Treatment for Xylocis

XML Treatment for Xylocis tortilicornis

XML Treatment for Apatini

XML Treatment for Phonapate

XML Treatment for Phonapate fimbriata

XML Treatment for Lyctinae

XML Treatment for Lyctini

XML Treatment for Lyctus

XML Treatment for Lyctus africanus

XML Treatment for Lyctoxylon

XML Treatment for Lyctoxylon dentatum

XML Treatment for Minthea

XML Treatment for Minthea bivestita

XML Treatment for Trogoxylini

XML Treatment for Trogoxylon

XML Treatment for Trogoxylon impressum

XML Treatment for Trogoxylon spinifrons

XML Treatment for Polycaoninae

XML Treatment for Melalgus

XML Treatment for Melalgus batillus

XML Treatment for Psoinae

XML Treatment for Coccographis

XML Treatment for Coccographis nigrorubra

## Figures and Tables

**Figure 3a.**
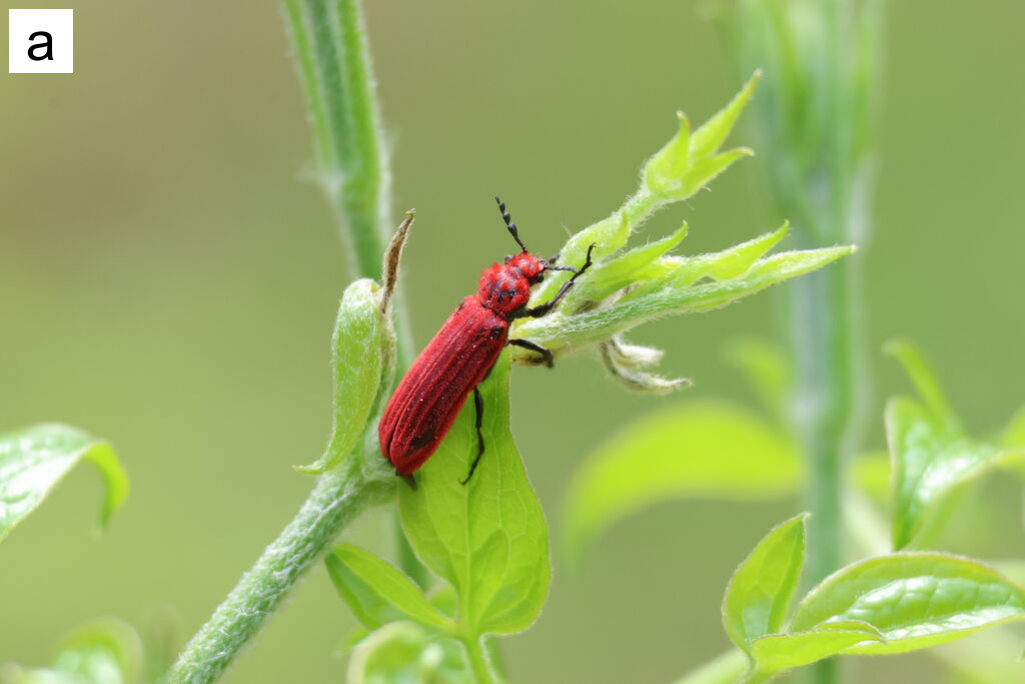
Lateral view (photo by Wen-Qi Zhu);

**Figure 3b.**
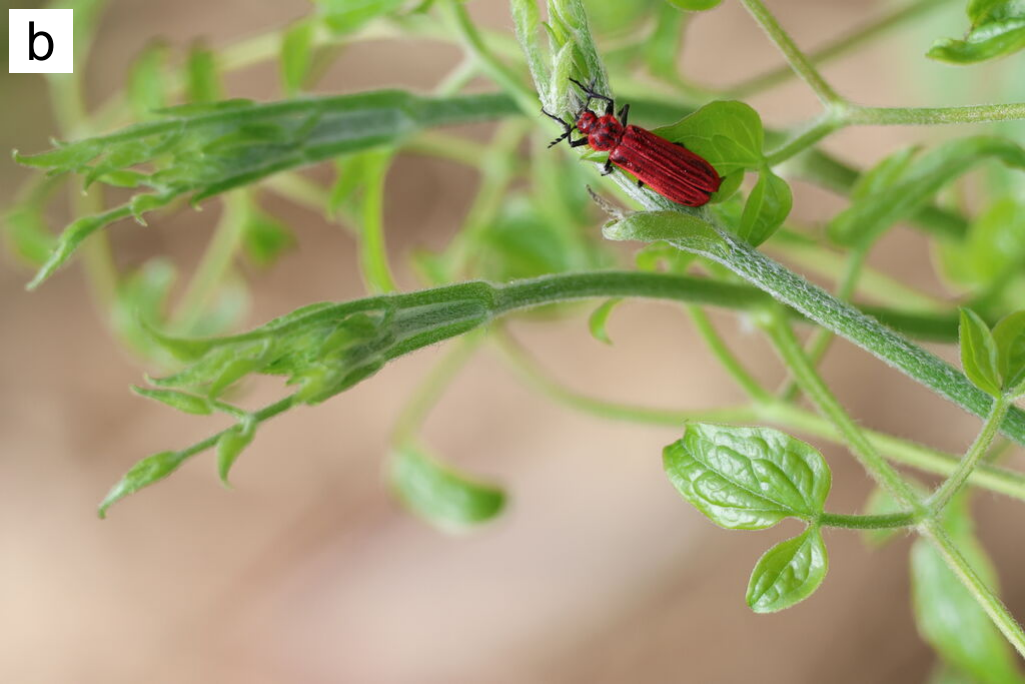
Dorsal view (photo by Jia-Min Chen).
